# Association between Parathyroid Hormone, 25 (OH) Vitamin D, and Chronic Kidney Disease: A Population-Based Study

**DOI:** 10.1155/2017/7435657

**Published:** 2017-03-07

**Authors:** Wei-Hao Wang, Li-Wei Chen, Chin-Chan Lee, Chiao-Yin Sun, Yu-Chiau Shyu, Heng-Rong Hsu, Rong-Nang Chien, I-Wen Wu

**Affiliations:** ^1^Department of Nephrology, Keelung Chang Gung Memorial Hospital, Keelung, Taiwan; ^2^Department of Gastroenterology and Hepatology, Keelung Chang Gung Memorial Hospital, Keelung, Taiwan; ^3^College of Medicine, Chang Gung University, Taoyuan, Taiwan; ^4^Community Medicine Research Center, Keelung Chang Gung Memorial Hospital, Keelung, Taiwan

## Abstract

Identification of the accurate risk factor for CKD remains mandatory to combat the high prevalence of diseases. Growing evidence suggests the association of serum vitamin D with diverse health conditions. However, the relationship between vitamin D, intact parathyroid hormone (PTH), and calcium-phosphate metabolism and development of CKD remains controversial. We conduct this cross-sectional observational study to investigate the association between serum 25 (OH) vitamin D, intact PTH, and calcium and phosphate levels with eGFR and albuminuria, as a surrogate marker of CKD, in a community population. A total of 4080 participants were recruited. The mean age was 58.4 ± 13.3 years and 1480 (36.3%) were men. The mean eGFR was 94.1 ± 26.3 mL/min/1.73 m^2^. The prevalence of CKD was 19.8%. Serum 25 (OH) vitamin D and log intact PTH levels were inversely correlated with eGFR but positively correlated with log albuminuria. Logistic regression analysis identified the log intact PTH as an independent factor associated with eGFR ≤ 60 mL/min/1.73 m^2^ and proteinuria. This association was consistent when serum intact PTH was analyzed as continuous as well as categorical variables (as hyperparathyroidism). The relationship remains significant using resampling subset analysis with comparable baseline characteristics and adjustment for 25 (OH) vitamin D, calcium, and phosphate levels. This finding warranted further research to clarify the causal relationship of PTH/25 (OH) vitamin D with the risk of CKD in the general population.

## 1. Introduction

Chronic kidney disease (CKD) is a worldwide public health issue because of its wide association with multiple comorbidities, demanding high cardiovascular events, mortality, and expensive medical cost. The worldwide prevalence of CKD ranged from 8 to 16%, dependent on the disease definition, study design, and racial groups [1]. The estimated national prevalence of CKD is 11.9% [2] in Taiwan, which has the highest incidence and prevalence of end-stage renal disease (ESRD) worldwide [3]. Ascertainment of accurate risk factors associated with CKD is mandatory to allow timely diagnosis and intervention to decrease the burden of the disease. The associations of CKD with several traditional risk factors (such as hypertension, diabetes, obesity, smoking, dyslipidemia, and metabolic syndrome) were well established in the literature [4]. Epidemiological literatures emphasized the association between serum vitamin D level and nonskeletal disease as cardiovascular events [5–7], metabolic syndrome [8], cancer [9, 10], autoimmune disease [11], and critical illness [12]. The decrease of glomerular filtration rate restricts delivery of substrate to the 1-alpha-hydroxylase and decreases the production of 1,25 (OH) vitamin D by the kidney in CKD patients. The interplay between vitamin D, fibroblast growth factor-23 (FGF-23), intact parathyroid hormone (PTH), and calcium-phosphorus-bone metabolism is disrupted with the progression of renal function [13–15]. However, the relationship between vitamin D, intact PTH, and calcium-phosphate metabolism and development of CKD remains controversial.

A Korean population-based study found a biphasic change of serum vitamin D levels, according to CKD severity. The estimated glomerular filtration rate (eGFR) was negatively associated with serum vitamin D levels [16] in the entire population; however, the mean vitamin D values were decreased with the lowering of eGFR levels in moderate and severe CKD stages [16, 17]. A Swiss population study did not find any association between serum vitamin D levels and CKD or albuminuria [18]. In addition, the association between vitamin D levels and renal function decline was lost after adjustment for baseline eGFR [19]. Accumulating evidence indicated the association of abnormal calcium-phosphate metabolism with cardiovascular disease in the general population. Intact PTH levels were associated with hypertension [20] and abnormal calcium-phosphate metabolism was associated with coronary artery calcification [21]. The prevalence ratio of intact PTH among participants of the US National Health and Nutrition Examination Survey showed a stepwise increment of 2.30 and 4.69 for individuals with an eGFR of 45 to 59 and 30 to 44 mL/min/1.73 m^2^ compared with those people having eGFR > or = 60 mL/min/1.73 m^2^ [22]. While the serum value variation and the relationship between vitamin D/intact PTH and hard outcomes were evident in moderate to late stage CKD and ESRD patients, little is known about the exact role of vitamin D, intact PTH, and calcium and phosphate levels all together in the risk of CKD in healthy subjects.

We conduct this cross-sectional observational study to investigate the association between serum 25 (OH) vitamin D, intact PTH, and calcium and phosphate levels with eGFR and albuminuria and the risk of CKD in a community population.

## 2. Method

### 2.1. Patient Setting and Data Description

Participants of community health activity from August 2013 to May 2016 in the northeastern region of Taiwan were enrolled in the study. Participants aged greater than 30 years and who were not pregnant were included after obtaining informed consent (*n* = 4916). Individuals who had received vitamin D supplementation or any over-the-counter vitamin supplements (*n* = 830) or incomplete baseline biochemistry data (*n* = 6) were excluded from the analysis. Demographic data were assessed from questionnaires. Anthropometric and biochemistry measurements were performed at the entry of the study. Blood samples were obtained after an overnight fast, and the following parameters were determined: complete blood cell count, liver and renal biochemistry parameters, lipid profiles, fasting sugar, and intact PTH and total 25 (OH) vitamin D levels. This study was approved by the ethics committee of the institutional review board of the Keelung Chang Gung Memorial Hospital.

### 2.2. Definitions

CKD were defined by the National Kidney Foundation: K/DOQI classification for CKD and were determined as having persistent proteinuria or a decreased eGFR of less than 60 mL/min/1.73 m2, determined by the abbreviated Modification of Diet in Renal Disease equation [23]. Serum Cr was assessed by spectrophotometric analysis using a modified kinetic Jaffe reaction with standardization of the creatinine calibration to an isotope dilution mass spectrometry reference measurement procedure. Proteinuria was determined if urine albumin-to-creatinine ratio > 30 g/g or urine protein-to-creatinine ratio > 150 g/g. Diabetes mellitus was defined as a fasting glucose level ≥ 126 mg/dL or use of any hypoglycemic medication. Hypertension was considered present if the patient received medical therapy for such a condition or if blood pressure was >140/90 mmHg. Hypercholesterolemia was defined as a total cholesterol level ≥ 200 mg/dL. Smoking and alcohol drinking indicated any sustained past or current behaviors. Metabolic syndrome was defined according to the Adult Treatment Panel III criteria as the presence of at least three of the following five traits: visceral (abdominal) obesity, determined on the basis of the Asian waist circumference cut-offs (men: >90 cm, women: >80 cm); blood pressure > 130/85 mmHg or drug treatment for essential hypertension; serum high-density lipoprotein cholesterol (HDL-C) level <40 mg/dL (1 mmol/L) in men and <50 mg/dL (1.3 mmol/L) in women or drug treatment for low HDL-C; serum triglycerides (TG) level > 150 mg/dL (1.7 mmol/L) or drug treatment for elevated TG; and fasting plasma glucose level >100 mg/dL (5.6 mmol/L) or drug treatment for DM. Obesity was evaluated according to the WHO classification as having a body mass index of 30 kg/m2 or more [24]. Serum concentrations of 25 (OH) vitamin D were measured using a radioimmunoassay (Vitamin D Total, Roche Diagnostics, Mannheim, Germany) according to the manufacturer's instructions. Vitamin D status was defined as “deficient” (<20 ng/mL), “insufficient” (20–30 ng/mL), and “sufficient” (>30 ng/mL) [25]. Hypocalcemia was defined as corrected Ca less than 8.5 mg/dL; hyperphosphatemia, *p* greater than 4.5 mg/dL, or phosphate binder use and hyperparathyroidism, intact parathyroid hormone more than twice the upper limit of normal, corresponding to 70 pg/mL [26]. Because of the cross-sectional nature of the study and to avoid misdiagnosis of CKD, we used two surrogate indices of CKD (eGFR < 60 mL/min and proteinuria) to establish the association between the biomarker of interest and the outcome.

### 2.3. Statistical Methods

Descriptive statistics were expressed as mean ± standard deviation, median, range, or percentage frequency, as appropriate. All variables were tested for normal distribution by Kolmogorov-Smirnov test. Data were log-transformed to approximate normal distribution. Student's* t*-test or Mann–Whitney* U* test was applied to compare the mean of continuous variables. Categorical data were tested using the Chi-square test. Pearson or Spearman correlation coefficients were appropriately used to test the correlation between serum 25 (OH) vitamin D, intact PTH, and calcium and phosphate levels with eGFR or proteinuria. Logistic regression analysis was applied to identify the association between these variables with the outcome of interest, after adjusting for potential confounders, such as age or gender. Conditional logistic regression analysis was performed to evaluate the odd ratio of factors associated with outcome in the resampling subset, matched by age (±1 year old), gender, CKD stage, presence of hypertension, and metabolic syndrome, at 1 : 2 ratio. All statistical tests were two-tailed, and a *p* < 0.05 was considered statistically significant. Data were analyzed using SPSS 17.0 for Windows (SPSS Inc., Chicago, IL).

## 3. Results

**Figure 1:**
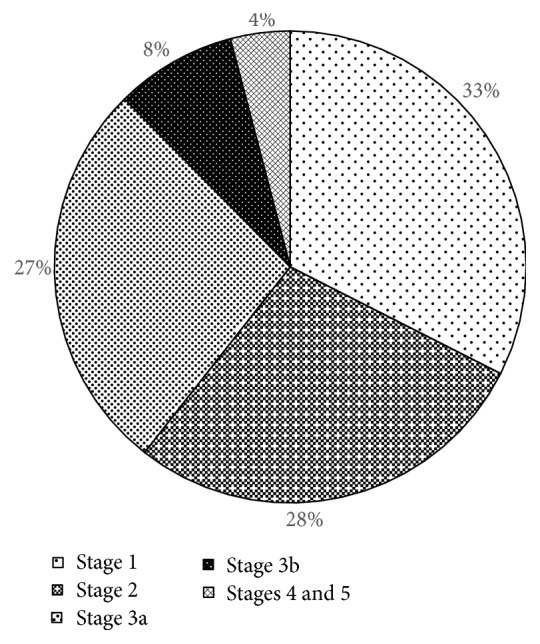
Distribution of CKD stage of the entire population.

**Figure 2:**
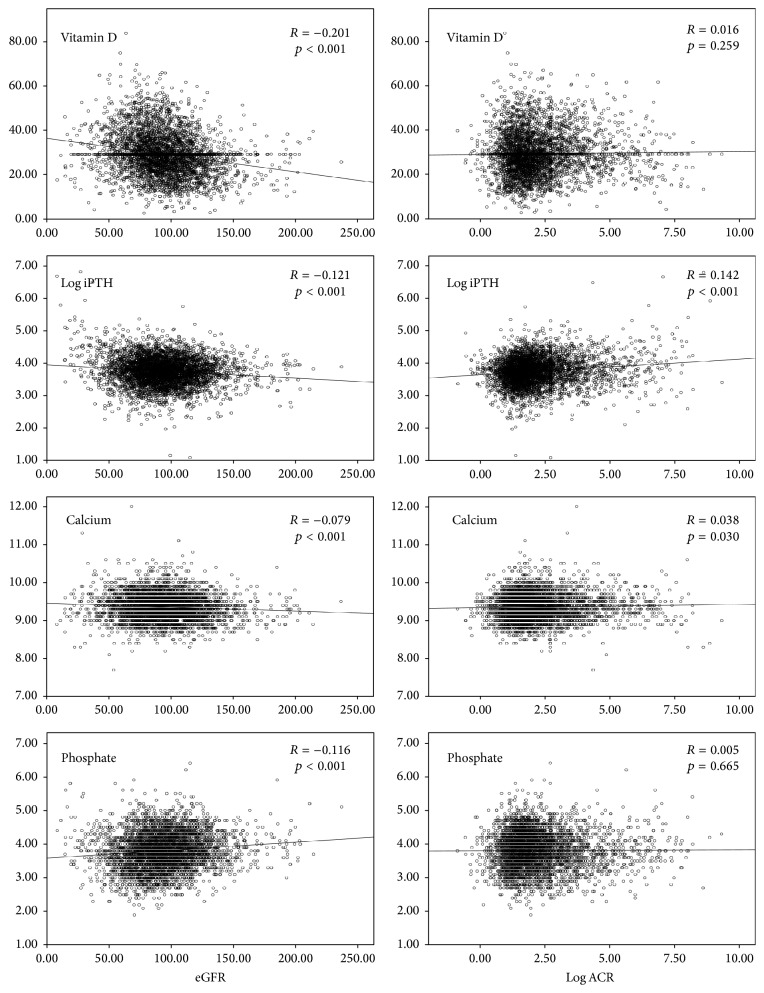
Correlation of serum 25 (OH) vitamin D, log intact PTH, and calcium and phosphate with eGFR and log albuminuria-to-creatinine ratio.

**Figure 3:**
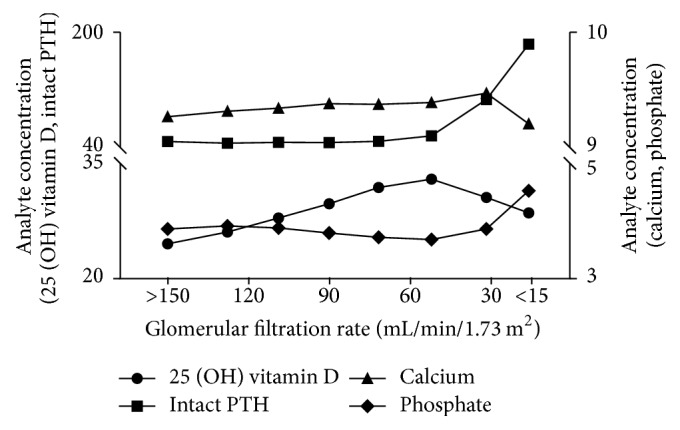
Trajectory of serum 25 (OH) vitamin D, intact PTH, and calcium and phosphate with eGFR.

A total of 4080 participants were included in the analysis. The mean age was 58.4 ± 13.3 years and 1480 (36.3%) were men. The mean eGFR of the study population was 94.1 ± 26.3 mL/min/1.73 m^2^.  Table 1 lists the baseline characteristics of all participants and between two groups. The overall prevalence of CKD was 19.8%.  Figure 1 illustrates the distribution of CKD stage of the entire population. CKD patients were more likely to be older, to be male, and to be obese. They were more likely to have diabetes, hypertension, metabolic syndrome, and smoking/drinking habits. CKD patients had higher serum 25 (OH) vitamin D [31.1 ± 10.5 ug/mL versus 29.0 ± 9.0 ug/mL, *p* < 0.001] and higher intact PTH [46.3 (8.3, 898.9) pmol/L versus 42.2 (3.0, 311.2) pmol/L, *p* < 0.001] levels than normals (Table 1).  Table 2 summarizes serum 25 (OH) vitamin D status stratified by eGFR levels and albuminuria-to-creatinine ratio. Serum 25 (OH) vitamin D (*r* = −0.212, *p* < 0.001), log intact PTH (*r* = −0.121, *p* < 0.001), and calcium (*r* = −0.079, *p* < 0.001) were inversely correlated with eGFR, while phosphate (*r* = 0.116, *p* < 0.001) was positively correlated with eGFR. However, serum 25 (OH) vitamin D (*r* = 0.016, *p* = 0.259), log intact PTH (*r* = 0.142, *p* < 0.001), calcium (*r* = 0.038, *p* = 0.030), and phosphate (*r* = 0.005, *p* = 0.665) were positively correlated with log albuminuria-to-creatinine ratio (Figure 2).  Figure 3 depicts the trajectory of these biomarkers with eGFR. Logistic regression analysis identified the log intact PTH as an independent factor associated with CKD [crude: odd ratios (OR), 1.985; 95% confidential interval (CI), 1.663–2.371, *p* < 0.001; model 1 (adjusted for age and gender): OR, 1.618; 95% CI, 1.345–1.948, *p* < 0.001; model 2 (adjusted for age, gender, 25 (OH) vitamin D, calcium, and phosphate): OR, 1.796, 95% CI, 1.479–2.181, *p* < 0.001]. The association of intact PTH with CKD was consistent when assessed in a categorical fashion, as hyperphosphatemia [crude: OR, 2.530; 95% CI, 2.057–3.112, *p* < 0.001; model 1 (adjusted for age and gender): OR, 2.074; 95% CI, 1.662–2.589, *p* < 0.001; model 2 (adjusted for age, gender, 25 (OH) vitamin D status, hypocalcemia, and hyperphosphatemia): OR, 2.128, 95% CI, 1.699–2.667, *p* < 0.001 (Table 3)]. Again, log intact PTH was an independent factor associated with proteinuria [crude: OR, 1.744, 95% CI, 1.442–2.109, *p* < 0.001; model 1 (adjusted for age and gender): OR, 1.477, 95% CI, 1.217–1.793, *p* < 0.001; model 2 (adjusted for age, gender, 25 (OH) vitamin D status, hypocalcemia, and hyperphosphatemia): OR, 1.579, 95% CI, 1.291–1.932, *p* < 0.001]. The association of intact PTH with proteinuria was consistent when assessed in a categorical fashion, as hyperphosphatemia [crude: OR, 2.212, 95% CI, 1.770–2.766, *p* < 0.001; model 1 (adjusted for age and gender): OR, 1.865, 95% CI, 1.480–2.351, *p* < 0.001; model 2 (adjusted for age, gender, 25 (OH) vitamin D status, hypocalcemia, and hyperphosphatemia): OR, 1.895, 95% CI, 1.497–2.398, *p* < 0.001 (Table 4)]. The associations of serum 25 (OH) vitamin D, calcium, and phosphate levels with CKD or proteinuria were not significant after adjusting for confounder or analysis in a categorical manner (Tables 3 and 4).

To control as much as possible the confounding effect of baseline characteristics of patients on the outcome of study, we have resampled a subset of patients from stratified sampling by CKD stage with individualized match to age, gender, presence of hypertension, or metabolic syndrome. Stratified sampling by CKD stage identified 464 CKD patients with their matched counterparts (*n* = 928 normals). Only 1392 patients were included in the subset analysis after individualized matched pair of ±1 y/o of age. The mean age of resampling subset was 61.0 ± 10.9 years and 39% of them were men. Hypertension was present in 73% and metabolic syndrome was present in 43% of the resampling population. Both the log intact PTH and hyperparathyroidism remained a significant risk factor for eGFR ≤ 60 mL/min and albuminuria, after adjustment for age, gender, serum 25 (OH) vitamin D, and calcium or phosphate levels (Table 5).

## 4. Discussion

This community-based study found that the serum PTH levels were inversely correlated with eGFR but positively correlated with albuminuria. Either the increase of serum PTH levels or the presence of hyperparathyroidism was independently associated with the risk of CKD, after adjustments for confounders or by using the resampling subset of comparable baseline characteristics. The serum PTH levels increased in the early stage of CKD, before the change of 25 (OH) vitamin D-calcium-phosphate axis, and may serve as a potential risk factor for CKD.

Optimal serum levels of vitamin D in healthy subjects and CKD patients are not well understood. Growing evidence indicated the relationship between abnormal vitamin D levels and metabolic syndrome, diabetes, cancer, and chronic diseases; however, the association of vitamin D and the risk of CKD remains controversial [27]. Our findings that serum 25 (OH) vitamin D levels increased with the decline of eGFR and the insignificant association with CKD are consistent with some studies [16, 18, 19], but not with others [28, 29]. Guessous et al. found that the prevalence of serum vitamin D concentration and deficiency status were similar in CKD and non-CKD subjects in a cross-sectional Swiss population-based study [18]. They did not find any significant association of serum vitamin D levels with incident CKD, incident albuminuria, or rapid renal function decline, after a mean follow-up of 5.5 years [19]. On the other hand, findings from the 10-year Prevention of Renal and Vascular End-Stage Disease prospective cohort demonstrated that low plasma vitamin D was associated with the risk of developing albuminuria rather than reduced eGFR in high sodium intake individuals [28]. However, investigation on normal renal function subjects found that high serum vitamin D levels were independently associated with high serum creatinine and recalled the potential role of muscle size as a contributing factor to this elevation [29]. Several factors can influence serum levels of vitamin D, including analytic method, seasonal variation, latitude, air pollution, sun exposure, and physical activity [30]. Furthermore, meta-analyses to evaluate the concentration of vitamin D and health outcome did not show convincing data for the association of this biomarker with renal outcome in healthy or CKD patients [27].

The finding that serum PTH levels and hyperparathyroidism were independent risk factors for CKD and its surrogates (eGFR and proteinuria) was novel. Molecularly, parathyroid hormone is considered as procalcific and profibrotic through promoting mRNA and protein expression of the receptor of advanced glycation end products (RAGE) and interleukin 6, enhancing abnormal calcium-phosphate homeostasis and renin-angiotensinogen-aldosterone hyperactivity; even some have advocated its possible roles as cardiovascular or uremic toxins [31–33]. On the other hand, treatment of teriparatide (human PTH) in low-density lipoprotein receptor −/− mice exhibited induction of osseous osteopontin expression and serum osteopontin levels, indicating inhibition of vascular calcification and aortic osteogenic differentiation. The study suggested possible beneficial actions of PTH at early stages of macrovascular disease in responses to diabetes and dyslipidemia [34]. Clinically, a Swedish population-based study showed that normocalcemic, vitamin D sufficient hyperparathyroidism was common and indolent in a long-term follow-up of 17 years. This condition triggered low morbidities and had no association with creatinine at follow-up [35]. However, data of the Germany Calcific Uraemic Arteriolopathy Registry indicated development of calciphylaxis in dialysis patients with mean PTH levels of 147 (IQR: 72–276) pg/mL [36]. In spite of the promising results of our study, caution in the interpretation of PTH as a powerful biomarker should be taken into account, including intermethod and interpersonal variability, target metabolite to measure, vitamin D status, baseline renal function, age, gender, menopausal status, body mass index, race, and end-organ hyporesponsiveness [37–40]. Further studies are needed to demonstrate the exact relationship between high intact PTH and development of CKD in the general population.

A peculiar finding of the present study was the elevated odd ratio of having eGFR < 60 mL/min associated with serum calcium levels, even after considering confounders. However, this association was no longer existent when the serum calcium level was assessed in a categorical manner. Physiologically, serum calcium levels decrease with the decline of eGFR. This phenomenon appears in the late stage of CKD and contributes in part to the hyperparathyroidism cascade leading to the development of CKD-bone mineral disease and adverse patient outcomes. Conversely, high serum calcium levels promote neointimal calcification and increase cardiovascular events and mortality. The paradoxical role of serum calcium levels was rarely explored in healthy or early stage CKD patients and is unclear. Association of high corrected serum calcium with both the number of the metabolic syndrome components and the number of nonconventional cardiometabolic risk factors (uric acid, homocystein, and gamma-glutamyltransferase) was observed in Caucasian people, independently of the metabolic syndrome and body mass index [41]. A French multicentric study found that increased serum calcium concentration was independently and positively associated with high pulse pressure and hypertension [42], an important risk factor for CKD. Putting all these lines of evidence together, the exact impact of serum calcium levels on the renal functions of healthy people deserves further study.

The strength of our study was in the use of a large number of participants with high range for renal function, simultaneous measurement of biochemistry parameters that minimize bias in the variability of analytic testing, and exclusion of exogenous vitamin D use. The use of subset sampling analysis of comparable characteristics has avoided confounding effects from baseline data on the study outcome. However, some limitations of study should be addressed. First, findings from the cross-sectional design study should not be interpreted in casual terms. Second, all participants of the study came from the northeastern region of Taiwan (Keelung and its neighboring areas) and had unique geographic (latitude, 25°N), cultural, and dietary characteristics that limited generalizability of the present findings to other populations. Finally, important drivers of renal calcium-phosphorus handling, such as FGF-23 and Klotho, were not measured in the present study. Primary hyperparathyroidism could not be excluded completely, because parathyroid images were not available. Universal consensus on the target fragments, analytic method, and reference value of measurement for these two biomarkers is still in debate. Further investigation should be warranted to elucidate the interplay of FGF-23, Klotho, vitamin D, and parathyroid axis with the risk of CKD.

In conclusion, this community-based study confirmed the association of serum levels of PTH and hyperparathyroidism with the risk of CKD. The serum levels of PTH increased in parallel with albuminuria but inversely with eGFR. Using a sample of the general Taiwanese population, the current study could provide a description of the prevalence of elevated serum intact PTH levels across a broad spectrum of kidney functions. This significant association with low eGFR was independent of dietary intake of calcium and serum levels of calcium, phosphorus, and 25 (OH) D. Given its association with adverse outcomes in CKD, reducing serum intact PTH levels may be an important goal for improving CKD-MBD and may be potentially helpful for CKD prevention. The findings of the present study offer an insight for a further well-designed prospective study to clarify the causal relationship of PTH and 25 (OH) vitamin D with renal function and risk of CKD in the general population.

## Figures and Tables

**Table 1:** Demographic characteristics of all patients and stratified by CKD (*n* = 4080).

	All (*n* = 4080)	Non-CKD (*n* = 3273)	CKD (*n* = 807)	*p*
Age, years	58.4 ± 13.3	56.5 ± 12.6	66.1 ± 12.9	<0.001
Male, number (%)	1480 (36.3%)	1134 (34.6%)	346 (42.9%)	<0.001
Diabetes, number (%)	722 (17.7%)	410 (12.5%)	312 (38.7%)	<0.001
Hypertension, number (%)	2197 (53.8%)	1607 (49.3%)	590 (73.2%)	<0.001
Metabolic syndrome, number (%)	1277 (31.3%)	863 (26.4%)	414 (51.3%)	<0.001
Obesity, number (%)	383 (9.4%)	264 (8.1%)	119 (14.7%)	<0.001
Physical activity, min/day	12.9 (0, 660)	12.9 (0, 660)	17.1 (0, 600)	0.137
Smoking, number (%)	1032 (25.3%)	806 (24.6%)	226 (28.0%)	0.048
Alcohol drinking, number (%)	642 (15.7%)	534 (16.3%)	108 (13.4%)	0.040
Body mass index, kg/m^2^	24.9 ± 3.8	24.6 ± 3.7	26.0 ± 3.8	<0.001
Systolic BP, mmHg	131.9 ± 19.4	129.6 ± 18.5	141.3 ± 20.1	<0.001
Diastolic BP, mmHg	78.5 ± 11.9	77.7 ± 11.5	81.9 ± 12.8	<0.001
Laboratory				
eGFR, mL/min per 1.73 m^2^ (MDRD)	94.1 ± 26.3	98.3 ± 22.6	77.1 ± 32.6	<0.001
BUN, mg/dL	13.7 ± 5.5	12.7 ± 3.7	17.5 ± 8.9	<0.001
Serum creatinine, mg/dL	0.8 ± 0.3	0.7 ± 0.2	1.0 ± 0.5	<0.001
Serum albumin, g/dL	4.7 ± 0.3	4.7 ± 0.3	4.6 ± 0.3	<0.001
Cholesterol, mg/dL	209.7 ± 39.2	210.1 ± 37.4	208.2 ± 45.8	0.209
Triglycerides, mg/dL	128.4 ± 114.4	121.4 ± 103.3	156.5 ± 148.1	<0.001
hs-CRP, mg/L	1.0 (0.2, 211.4)	0.9 (0.2, 104.1)	1.6 (0.2, 211.4)	<0.001
Urine albumin-to-creatinine ratio, g/g	6.5 (0.4, 11067.4)	5.4 (0.6, 29.8)	49.7 (0.4, 11067.4)	<0.001
25 (OH) vitamin D, ug/mL	29.4 ± 9.4	29.0 ± 9.0	31.1 ± 10.5	<0.001
iPTH, pmol/L	43.0 (3.0, 898.9)	42.2 (3.0, 311.2)	46.3 (8.3, 898.9)	<0.001
Serum calcium, mg/dL	9.4 ± 0.3	9.4 ± 0.3	9.4 ± 0.4	0.055
Corrected calcium, mg/dL	8.8 ± 0.3	8.8 ± 0.3	8.9 ± 0.4	<0.001
Serum phosphate, mg/dL	3.8 ± 0.5	3.8 ± 0.5	3.8 ± 0.6	0.003
25 (OH) vitamin D status				<0.001
Vitamin D sufficient, number (%)	1383 (33.9%)	1042 (31.8%)	341 (42.3%)	
Vitamin D deficient, number (%)	2124 (52.1%)	1755 (53.6%)	369 (45.7%)	
Vitamin D insufficient, number (%)	573 (14.0%)	476 (14.5%)	97 (12.0%)	

The values are expressed as means (SD) or median (min, max).

Corrected calcium = serum calcium + 0.8 *∗* (4 − serum albumin).

**Table 2:** Serum 25 (OH) vitamin D status stratified by eGFR and albuminuria-to-creatinine ratio.

Variable	Vitamin D sufficient, number (%)	Vitamin D deficient, number (%)	Vitamin D insufficient, number (%)	*p*
Mean eGFR, mL/min				<0.001
eGFR > 90 mL/min	607 (27.2%)	1227 (55.0%)	398 (17.8%)	
eGFR 90–60 mL/min	619 (40.5%)	772 (50.5%)	139 (9.1%)	
eGFR 60–45 mL/min	118 (54.1%)	79 (36.2%)	21 (9.6%)	
eGFR < 45 mL/min	39 (39.0%)	46 (46%)	15 (15%)	
Mean ACR ratio, g/g				0.2586
ACR < 30	1147 (32.8%)	1851 (53.0%)	497 (14.2%)	
ACR 30–300	203 (43.5%)	220 (47.1%)	44 (9.4%)	
ACR > 300	33 (28.0%)	53 (44.9%)	32 (27.1%)	

eGFR: estimated glomerular filtration rate; ACR: urine albumin-to-creatinine ratio.

**Table 3:** OR of variables associated with eGFR less than 60 mL/min.

Variable	Crude		Model 1		Model 2	
	OR (95% CI)	*p*	OR (95% CI)	*p*	OR (95% CI)	*p*
Continuous variables						
25 (OH) vitamin D, ug/mL	1.023 (1.015–1.031)	<0.001	1.001 (0.992–1.010)	0.795	1.005 (0.996–1.015)^*∗*^	0.276
Log intact PTH, pmol/L	1.985 (1.663–2.371)	<0.001	1.618 (1.345–1.948)	<0.001	1.796 (1.479–2.181)^*∗*^	<0.001
Calcium, mg/dL	1.276 (1.020–1.597)	0.033	1.475 (1.168–1.862)	0.001	1.617 (1.270–2.060)^*∗*^	<0.001
Phosphate, mg/dL	0.802 (0.694–0.926)	0.003	1.086 (0.919–1.283)	0.332	1.138 (0.959–1.350)^*∗*^	0.137
Categorical variables						
Vitamin D sufficient (yes versus no)	1		1		1	
Vitamin D deficient (yes versus no)	0.642 (0.544–0.758)	<0.001	0.872 (0.662–1.149)	0.330	0.959 (0.725–1.269)^#^	0.771
Vitamin D insufficient (yes versus no)	0.623 (0.485–0.800)	<0.001	0.726 (0.558–0.944)	0.017	0.777 (0.595–1.014)^#^	0.063
Hyperparathyroidism (yes versus no)	2.530 (2.057–3.112)	<0.001	2.074 (1.662–2.589)	<0.001	2.128 (1.699–2.667)^#^	<0.001
Hypocalcemia (yes versus no)	1.236 (0.529–2.892)	0.624	0.940 (0.374–2.360)	0.896	0.659 (0.261–1.665)^#^	0.378
Hyperphosphatemia (yes versus no)	0.935 (0.728–1.200)	0.598	1.237 (0.948–1.612)	0.117	1.298 (0.994–1.696)^#^	0.560

OR: odd ratios; CI: confidence interval.

Model 1: adjusted for age and gender.

Model 2:  ^*∗*^adjusted for age, gender, and all other continuous variables;  ^#^adjusted for age, gender, and all other categorical variables.

**Table 4:** OR of variables associated with proteinuria.

Variable	Crude		Model 1		Model 2	
	OR (95% CI)	*p*	OR (95% CI)	*p*	OR (95% CI)	*p*
Continuous variables						
25 (OH) vitamin D, ug/mL	1.016 (1.008–1.025)	<0.001	1.000 (0.991–1.010)	0.941	1.004 (0.994–1.014)^*∗*^	0.457
Log intact PTH, pmol/L	1.744 (1.442–2.109)	<0.001	1.477 (1.217–1.793)	<0.001	1.579 (1.291–1.932)^*∗*^	<0.001
Calcium, mg/dL	1.159 (0.907–1.481)	0.237	1.252 (0.980–1.599)	0.072	1.330 (1.037–1.705)^*∗*^	0.025
Phosphate, mg/dL	0.865 (0.740–1.012)	0.069	1.070 (0.897–1.276)	0.452	1.118 (0.935–1.338)^*∗*^	0.222
Categorical variables						
Vitamin D sufficient (yes versus no)	1		1		1	
Vitamin D deficient (yes versus no)	1.386 (1.060–1.813)	0.017	0.887 (0.666–1.183)	0.416	0.963 (0.720–1.289)^#^	0.801
Vitamin D insufficient (yes versus no)	0.971 (0.747–1.263)	0.826	0.756 (0.576–0.992)	0.044	0.802 (0.609–1.056)^#^	0.115
Hyperparathyroidism (yes versus no)	2.212 (1.770–2.766)	<0.001	1.865 (1.480–2.351)	<0.001	1.895 (1.497–2.398)^#^	<0.001
Hypocalcemia (yes versus no)	1.332 (0.542–3.272)	0.532	1.109 (0.436–2.821)	0.827	0.821 (0.320–2.107)^#^	0.682
Hyperphosphatemia (yes versus no)	0.992 (0.759–1.298)	0.955	1.201 (0.909–1.587)	0.198	1.249 (0.943–1.653)^#^	0.120

OR: odd ratios; CI: confidence interval.

Model 1: adjusted for age and gender.

Model 2:  ^*∗*^adjusted for age, gender, and all other continuous variables;  ^#^adjusted for age, gender, and all other categorical variables.

**Table 5:** Conditional logistic regression analysis in resampling subset of patients.

Variable	eGFR ≤ 60 mL/min/1.73 m^2^		Proteinuria	
	Adjusted OR (95% CI)	*p*	Adjusted OR (95% CI)	*p*
Continuous variables				
25 (OH) vitamin D, ug/mL	1.012 (0.999–1.025)^*∗*^	0.079	1.006 (0.993–1.020)^*∗*^	0.380
Log intact PTH, pmol/L	1.613 (1.240–2.099)^*∗*^	<0.001	1.471 (1.117–1.938)^*∗*^	0.006
Calcium, mg/dL	1.421 (1.003–2.014)^*∗*^	0.048	1.044 (0.722–1.510)^*∗*^	0.818
Phosphate, mg/dL	1.155 (0.913–1.463)^*∗*^	0.23	1.231 (0.962–1.576)^*∗*^	0.098
Categorical variables				
Vitamin D sufficient (yes versus no)	1		1	
Vitamin D deficient (yes versus no)	1.323 (0.896–1.953)^#^	0.16	1.096 (0.735–1.634)^#^	0.653
Vitamin D insufficient (yes versus no)	0.989 (0.685–1.427)^#^	0.951	0.887 (0.611–1.290)^#^	0.531
Hyperparathyroidism (yes versus no)	1.994 (1.442–2.758)^#^	<0.001	1.836 (1.313–2.567)^#^	<0.001
Hypocalcemia (yes versus no)	0.167 (0.020–1.373)^#^	0.096	0.232 (0.028–1.920)^#^	0.176
Hyperphosphatemia (yes versus no)	1.677 (1.162–2.421)^#^	0.006	1.611 (1.105–2.347)^#^	0.013

OR: odd ratios; CI: confidence interval; eGFR: estimated glomerular filtration rate.

^*∗*^Adjusted for age, gender, and all other continuous variables. ^#^Adjusted for age, gender, and all other categorical variables.
